# Unilateral pulmonary oedema caused by eccentric mitral regurgitation—multimodality evidence of mechanism and reversal after transcatheter edge-to-edge repair: a case report

**DOI:** 10.1093/ehjcr/ytag144

**Published:** 2026-03-03

**Authors:** Jun Yoshida, Goki Uno, Toraaki Okuyama, Takayuki Ogawa, Michifumi Tokuda

**Affiliations:** Division of Cardiology, Department of Internal Medicine, The Jikei University School of Medicine, 3-25-8 Nishi-Shinbashi, Minato-ku, Tokyo 105-8461, Japan; Division of Cardiology, Department of Internal Medicine, The Jikei University School of Medicine, 3-25-8 Nishi-Shinbashi, Minato-ku, Tokyo 105-8461, Japan; Division of Cardiology, Department of Internal Medicine, The Jikei University School of Medicine, 3-25-8 Nishi-Shinbashi, Minato-ku, Tokyo 105-8461, Japan; Division of Cardiology, Department of Internal Medicine, The Jikei University School of Medicine, 3-25-8 Nishi-Shinbashi, Minato-ku, Tokyo 105-8461, Japan; Division of Cardiology, Department of Internal Medicine, The Jikei University School of Medicine, 3-25-8 Nishi-Shinbashi, Minato-ku, Tokyo 105-8461, Japan

**Keywords:** Segmental pulmonary oedema, Pulmonary venous flow reversal, 3D transoesophageal echocardiography, Perfusion scintigraphy, Multimodal imaging, Case report

## Abstract

**Background:**

Unilateral pulmonary oedema is uncommon and frequently misattributed to pneumonia, delaying appropriate heart-failure care. Mechanistic clarification and demonstration of reversibility in a single patient can sharpen diagnostic reasoning and guide management.

**Case summary:**

An 88-year-old man presented with fever, inflammatory markers, and a right-upper-lobe ground-glass opacity. While pneumonia was suspected, transthoracic echocardiography revealed decompensated heart failure with severe mitral regurgitation (MR). Three-dimensional transoesophageal echocardiography (3D TEE) and contrast-enhanced computed tomography (CT) localized an eccentric MR jet towards the right superior pulmonary vein (RSPV). Pulmonary vein Doppler showed a vein-specific reversed systolic wave with diastolic extension in the RSPV, whereas the forward diastolic wave was preserved in other veins. After defervescence and partial decongestion, perfusion scintigraphy demonstrated segmental hypoperfusion matching the RSPV territory. Given high surgical risk, transcatheter edge-to-edge repair (TEER) was performed and reduced MR. After TEER, PV Doppler converted to forward systolic/diastolic waves, the chest radiograph cleared, and perfusion improved.

**Discussion:**

This case triangulates mechanism and reversibility by aligning morphologic (3D TEE/CT), haemodynamic (PV Doppler), and functional (perfusion scintigraphy) evidence, each demonstrated pre- and post-TEER. The multimodality concordance and TEER-demonstrated reversal strengthen causality beyond PV-flow-only reports and provide an actionable pathway for cases with unilateral opacities.

Learning points

- Unilateral or segmental opacities should warrant parallel evaluation for PV-directed MR, even when infection seems convincing.
- Concordant multimodality imaging (3D TEE/CT, PV Doppler, perfusion scintigraphy) can establish the mechanism of PV-directed MR causing unilateral pulmonary oedema.
- Pre/post-TEER changes demonstrated reversibility and strengthened the causal link between a PV-directed MR jet and unilateral pulmonary oedema.

## Introduction

Unilateral pulmonary oedema is uncommon, accounting for approximately two percent of cardiogenic cases, and is often misdiagnosed as pneumonia, which can delay appropriate heart failure care.^1^ In eccentric mitral regurgitation (MR), a right upper lobe (RUL) predominance is a classic yet under-recognized pattern.^1^

Omori *et al*. evaluated acute eccentric MR using pulmonary vein (PV) Doppler on transoesophageal echocardiography (TEE) and correlated vein-specific flow patterns with field-specific oedema severity. They showed that even when MR produces bilateral oedema, its distribution can be asymmetric.^2^ Other prior reports linked MR to unilateral oedema mainly via PV-flow assessment, without jet localization or functional perfusion imaging.^3,4^ In contrast, this case uniquely triangulates mechanism and reversibility by aligning morphologic [three-dimensional (3D) TEE/computed tomography (CT)], haemodynamic (PV Doppler), and functional (perfusion scintigraphy) evidence, all demonstrated before and after transcatheter edge-to-edge repair (TEER).

## Summary figure

**Figure:**
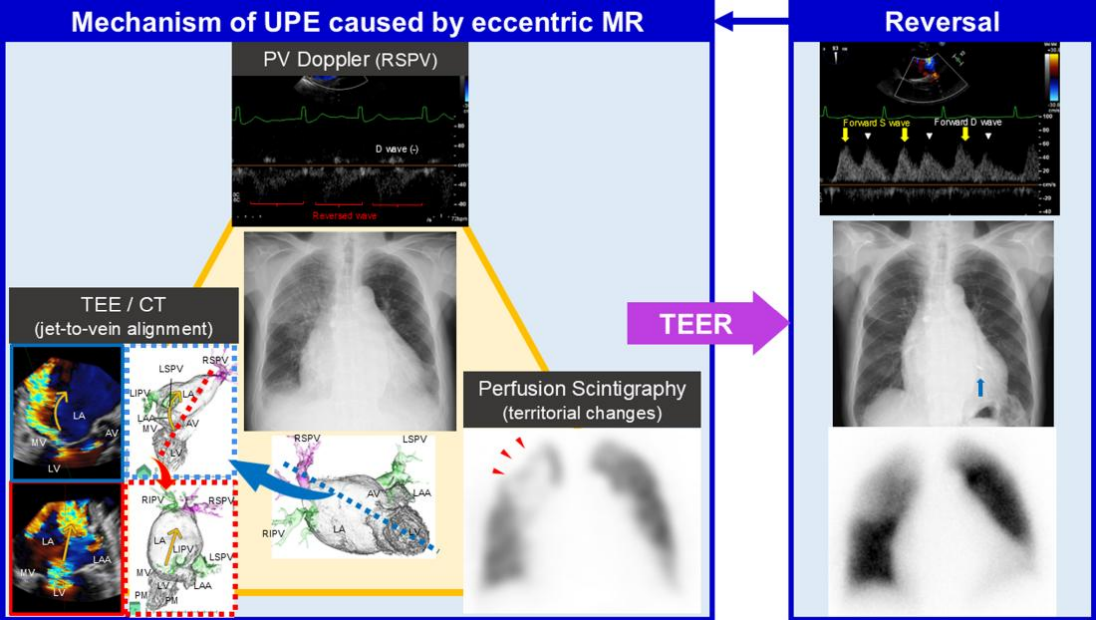


## Case presentation

An 88-year-old man with influenza A treated with oseltamivir became afebrile, but cough and new dyspnoea progressed, and chest radiography at the referring clinic showed RUL ground-glass opacity, prompting referral.

A physical examination revealed the following: Blood pressure, 132/92 mmHg; heart rate, 123 beats/min; body temperature, 37.9°C, and oxygen saturation of 92% on room air. A holosystolic murmur (Levine 2/6) was audible at the apex. Bilateral pitting oedema was present. His past medical history included bradyarrhythmic atrial fibrillation (no ablation), myelodysplastic syndrome.

Differential diagnoses included post-influenza viral or bacterial pneumonia, congestive heart failure with valvular disease, and PV stenosis.

We denote the index admission days as hospital day *N* (e.g. hospital Day 1). For the intervention admission, the procedure date is TEER Day 0 (TEER0); peri-procedural days are expressed as TEER−N/TEER+N.

**Figure 1:**
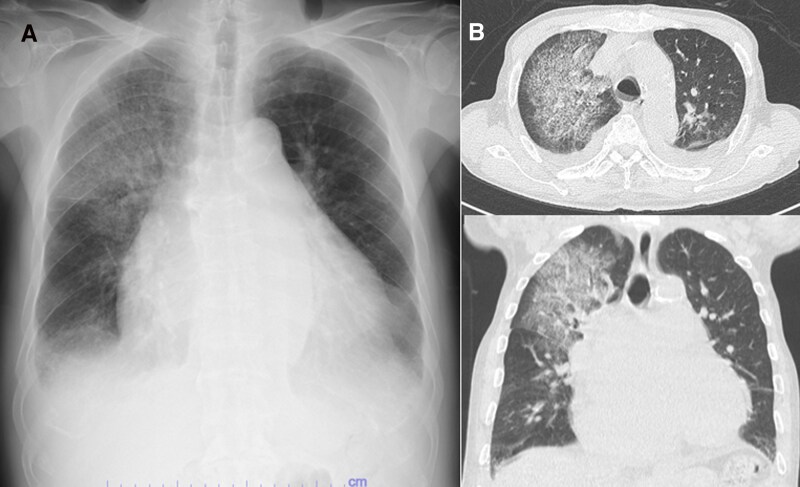
Chest radiograph and computed tomography findings on admission. (*A*) Chest radiography revealed marked cardiomegaly and extensive ground-glass opacity in the right upper lobe with partial consolidation. (*B*) Chest computed tomography revealed marked cardiomegaly with bilateral pleural effusions, extensive right upper lobe ground-glass opacity with partial consolidation, interlobular septal thickening, and relative subpleural sparing. The upper panel shows the axial view, and the lower panel shows the coronal view of the chest.

On hospital Day 1, his C-reactive protein and B-type natriuretic peptide were 15.19 mg/dL and 195.3 pg/mL, respectively. Chest radiography and CT on hospital Day 1 demonstrated cardiomegaly, effusions, ground-glass opacity with partial consolidation, interlobular septal thickening, and subpleural sparing (*Figure 1A* and *B*). Initial transthoracic echocardiography (TTE) on hospital day 1 revealed preserved left ventricular systolic function, severe MR, and severe tricuspid regurgitation (TR), with an estimated right ventricular systolic pressure of 78 mmHg, suggesting pneumonia with concomitant congestive heart failure related to valvular disease.

On hospital Day 1, ceftriaxone plus azithromycin were administered to treat pneumonia. Blood and sputum cultures were negative. For decongestion, intravenous diuretics were initiated and switched to oral diuretics. Despite defervescence and partial decongestion, the RUL opacity persisted, prompting consideration of organizing pneumonia and alveolar haemorrhage.

On hospital day 11, comprehensive TTE revealed severe MR due to A2 prolapse. Apical 4- and 3-chamber TTE with colour Doppler showed an eccentric MR jet tracking along the posterior and lateral left atrial wall (see Supplementary material online, *Videos S1A* and *S1B*) and a parasternal short-axis view at the mitral valve level demonstrated an MR jet originating from the A2 segment and directed towards the posterolateral left atrium (see Supplementary material online, *Video S1C*). Although TTE established eccentricity, jet-to-pulmonary-vein orientation could not be resolved. The left ventricular ejection fraction was 69% with LV end-diastolic/systolic diameters of 58/35 mm. The TR was moderate, and the estimated right ventricular systolic pressure was 63 mmHg. No findings were suggestive of infective endocarditis.

**Figure 2:**
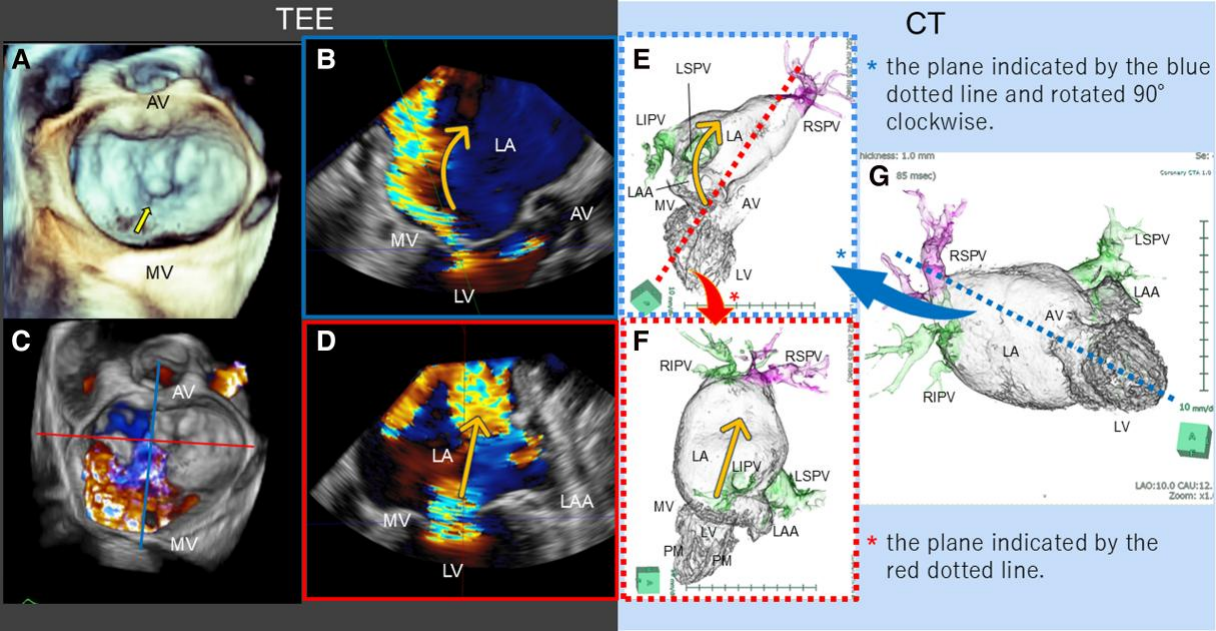
Anatomical relationship between the mitral regurgitation jet and the pulmonary veins on 3D transoesophageal echocardiography and contrast-enhanced computed tomography. (*A, C*) Three-dimensional transoesophageal echocardiography (3D transoesophageal echocardiography) with en face views of the mitral valve, showing A2 prolapse with chordal rupture (yellow arrow) (*A*) and eccentric severe mitral regurgitation (*C*). (*B, D*) 3D transoesophageal echocardiography colour Doppler of the mitral regurgitation jet due to A2 prolapse, displayed in the long-axis view (*B*) and commissure view (*D*). The long-axis view view (*B*) is obtained by sectioning along the blue line indicated on (*C*) (multiplanar reconstruction), and the commissure view (*D*) is obtained by sectioning along the red line on (*C*). Orange arrows indicate mitral regurgitation jet direction. (*G*) Volume-rendered reconstruction from contrast-enhanced computed tomography in left anterior oblique 10° and caudal 12° projections, depicting all four pulmonary veins, left-sided cardiac structures, and the aorta. The right superior pulmonary vein was depicted in purple, whereas the remaining pulmonary veins were depicted in green. (*E*) Multiplanar reconstruction along the blue dotted line in (*G*), rotated 90°clockwise. Orange arrows indicate mitral regurgitation jet direction. (*F*) Multiplanar reconstruction along the red dotted line in (*E*). Orange arrows indicate mitral regurgitation jet direction. *Panel correspondence and key finding*. Matched cross-sectional planes from 3D transoesophageal echocardiography colour Doppler and computed tomography multiplanar reconstruction are displayed side-by-side to demonstrate the jet direction. (*B*) corresponds to (*E*), and (*D*) corresponds to (*F*). Across these matched planes, the eccentric mitral regurgitation jet was directed towards the right superior pulmonary vein. AV, aortic valve; LA, left atrium; LV, left ventricle; LAA, left atrial appendage; LIPV, left inferior pulmonary vein; LSPV, left superior pulmonary vein; MV, mitral valve; RIPV, right inferior pulmonary vein; RSPV, right superior pulmonary vein; PM, papillary muscle.

**Figure 3:**
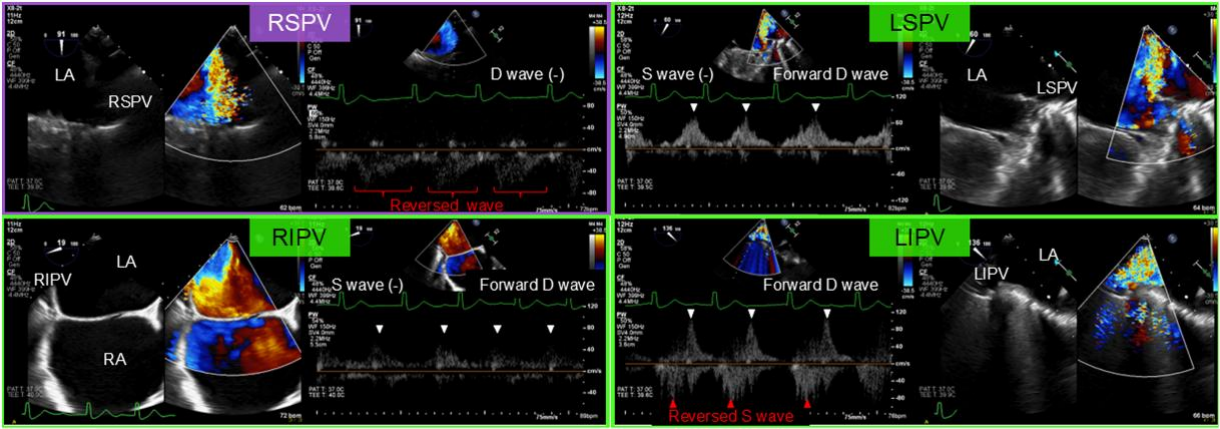
Flow patterns of each of the four pulmonary veins. While the other pulmonary veins showed reversed systolic (red arrowheads) or absent waves with forward diastolic waves (white arrowheads), the right superior pulmonary vein exhibited a reversed systolic wave with diastolic extension due to mitral regurgitation, with no distinct diastolic wave. D, diastolic; LA, left atrium; LIPV, left inferior pulmonary vein; LSPV, left superior pulmonary vein; RIPV, right inferior pulmonary vein; RSPV, right superior pulmonary vein; S, systolic.

On hospital day 13, 3D TEE identified severe MR due to A2 prolapse with chordal rupture; colour Doppler jet tracked along the posterior left atrial wall (*Figure2A–D*, Supplementary material online, *Video S2A*–*S2C*). Integrating 3D TEE with multiplanar reconstruction images from contrast-enhanced CT on hospital day 16, the MR jet was directed towards the right superior PV (RSPV) (*Figure2B* and *D–G*). Pulmonary vein flow patterns were as follows: RSPV (a reversed systolic wave extending into diastole with no distinct forward diastolic wave); other PVs (absent or reversed systolic wave with a forward diastolic wave) (*Figure3*).

**Figure 4:**
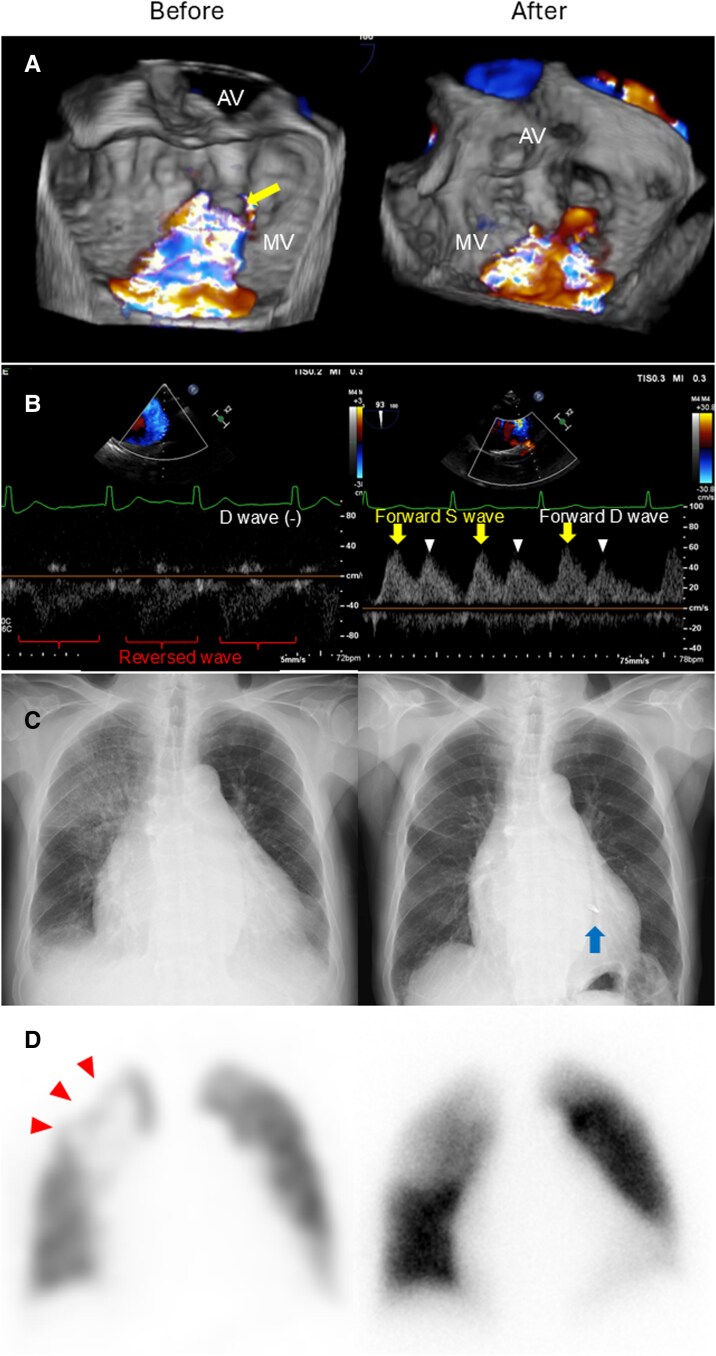
Changes in each imaging modality before and after the transcatheter edge-to-edge repair procedure. (*A*) The mitral regurgitation jet caused by A2 prolapse (left panel, yellow arrow) was divided into two jets by the clip, and its severity was reduced from grade 4+ to grade 2+ (right panel). (*B*) Pulmonary vein Doppler showing restoration of forward systolic (yellow arrows) and diastolic (white arrowheads) waves in the right superior pulmonary vein on the right panel. (*C*) Chest radiograph showing resolution of opacity in the right upper lobe. The clip is shown in the right panel (blue arrow). (*D*) Perfusion scintigraphy showing increased perfusion in the right upper lobe with improvement in the prior defect (red arrowheads). AV, aortic valve; D, diastolic; MV, mitral valve; S, systolic.

On hospital Day 14, perfusion scintigraphy was performed after defervescence and partial decongestion to distinguish residual inflammation from PV inflow-related malperfusion in the persistent RUL opacity. It demonstrated RUL segmental hypoperfusion with preserved aeration consistent with RSPV inflow impediment (*Figure4D*, left panel).

On hospital day 16, the chest radiograph remained unchanged, and contrast-enhanced CT demonstrated no focal narrowing of the PV ostia (see Supplementary material online, *Figure S1*). Thus, fixed PV stenosis was considered unlikely. Computed tomography lacked features suggestive of organizing pneumonia (peripheral/perilobular consolidation, reversed halo sign), and diffuse alveolar haemorrhage (diffuse or rapidly progressive ground-glass opacities with crazy-paving).

Given symptomatic heart failure due to MR and a high surgical risk, we planned TEER. The TR was managed conservatively. The patient was discharged on hospital Day 20 to await elective TEER 2 months later.

**Figure 5:**
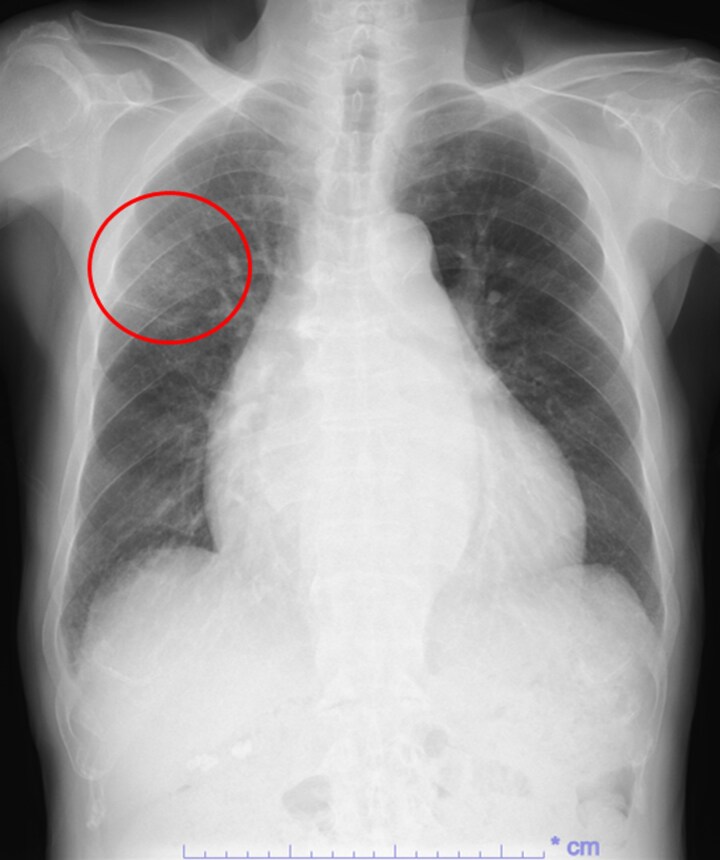
Chest radiograph five days before transcatheter edge-to-edge repair. Chest radiography shows slight residual opacity in the right upper lobe (red circle).

On TEER−5, chest radiography showed slight residual RUL opacity (*Figure5*); a C-reactive protein of ≤0.1 mg/dL. On TEER0, TEER was performed using the MitraClip system. One MitraClip XTW at A2/P2 reduced the MR from grade 4+ to grade 2+ (*Figure4A*). Immediately after the procedure, PV Doppler showed restoration of the systolic and diastolic forward waves in RSPV (*Figure4B*).

On TEER+5, TTE revealed moderate (grade 2+) MR. The right ventricular systolic pressure decreased to 46 mmHg.

On TEER+8, chest radiography showed improvement with resolution of RUL opacity (*Figure4C*, right panel). Perfusion scintigraphy showed increased perfusion in the RUL with improvement in the prior defect (*Figure4D*, right panel).

He was discharged uneventfully and remained free of heart-failure readmission at six months.

## Discussion

Unlike prior PV-flow–only reports, we provide concordant morphologic (3D TEE/CT), haemodynamic (PV Doppler), and functional (perfusion) evidence, all reversing after TEER.

In our case, systolic reversal with diastolic extension (biphasic reversal pattern) caused by eccentric MR jet from mitral valve prolapse was prominent only in the RSPV. We speculate that local left atrial overpressure plus jet inertia at the RSPV ostium may contribute to prolonged reverse flow into diastole, which is consistent with the biphasic reversal pattern. In contrast, the other PVs showed reversal limited to systole, with preserved forward diastolic waves. This vein-specific biphasic reversal of PV flow provided haemodynamic evidence for unilateral and upper-lobe-segmental oedema within the RSPV drainage territory. Beyond the PV flow, we visualized the anatomical relationship between the direction of the MR jet and the target PV using 3D TEE colour Doppler and contrast-enhanced CT. To obtain functional corroboration, lung perfusion scintigraphy, which provides functional imaging of regional pulmonary perfusion,^5^ was pursued after defervescence and partial decongestion because the RUL opacity persisted, and we sought to distinguish inflammatory consolidation from venous inflow-related malperfusion. The pattern (preserved aeration with segmental hypoperfusion concordant with the RSPV territory) raised diagnostic confidence that PV-directed MR, rather than residual pneumonia alone, was the dominant mechanism. By establishing a pre-intervention functional baseline of regional perfusion, we documented improvement after TEER and objectively demonstrated reversibility. These findings support a causal link between PV-directed MR and unilateral pulmonary oedema and may provide a clinical rationale for considering intervention in similar cases.

TEER was performed according to the guideline in the present case.^6^ After TEER, MR decreased, PV flow improved, with disappearance of the opacity on chest radiography and improvement of perfusion on scintigraphy observed. A prior TEER case of unilateral oedema due to acute MR from chordal rupture after myocardial infarction showed radiographic improvement only, without PV flow or mechanistic imaging.^7^ To our knowledge, no prior single-patient report has systematically demonstrated concordant pre- and post-TEER improvements across morphologic imaging (3D TEE and CT), PV haemodynamics, and functional perfusion imaging, which strongly supports MR as the cause of unilateral pulmonary oedema.

PV stenosis can cause lobar congestion.^8^ However, CT showed no ostial narrowing, PV Doppler favoured a jet-related mechanism, and findings reversed after TEER—arguing against fixed PV stenosis.

## Limitations

Ventilation scintigraphy was not performed; therefore, full ventilation–perfusion characterization was unavailable. The interval between initial imaging and TEER may have introduced temporal confounding. Nevertheless, the persistent opacity before TEER and concordant physiological/imaging reversal after TEER support a haemodynamic and reversible mechanism.

## Conclusion

Multimodal concordance and TEER-demonstrated reversal strengthen the causal link between PV-directed MR and unilateral oedema, offering an actionable pathway for unilateral opacities.

## Supplementary Material

ytag144_Supplementary_Data

## Data Availability

The data underlying this article are available from the corresponding author upon reasonable request.

## Lead author biography

Dr Jun Yoshida is a cardiologist specializing in cardiovascular imaging, with a focus on echocardiography. He graduated from the Jikei University School of Medicine in 2007 and was a Research Fellow in Echocardiography at Cedars-Sinai Medical Center (2017–2019). He currently serves in the Department of Cardiology at the Jikei University School of Medicine, where he became a lecturer in 2023. His clinical and research interests include valvular and structural heart disease, cardiomyopathies, and heart failure.

## Notes

**Consent:** Written informed consent was obtained from the patient in accordance with COPE guidelines.
