# Supplementary figures and images for: Unilateral pulmonary oedema caused by eccentric mitral regurgitation—multimodality evidence of mechanism and reversal after transcatheter edge-to-edge repair: a case report
Source: Eur Heart J Case Rep. 2026 Mar 3;10(3):ytag144. doi: 10.1093/ehjcr/ytag144 (PMC12998535; doi:10.1093/ehjcr/ytag144)

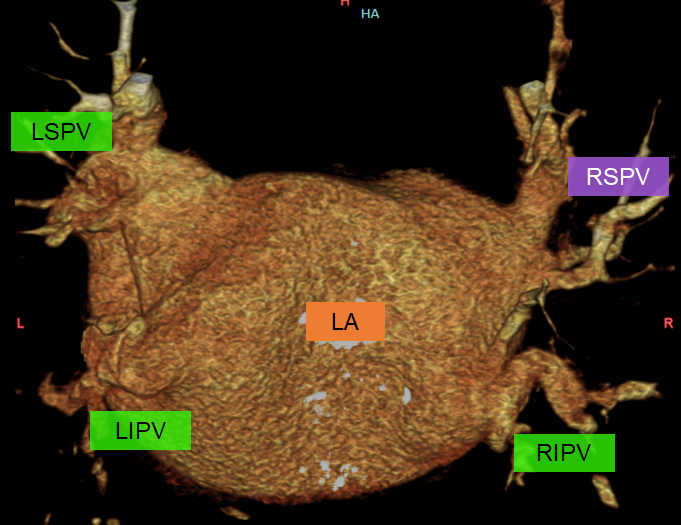

Supplement: ytag144_Supplementary_Data [file ytag144_supplementary_data.zip › Supplemental Figure 1.tif]
